# Multiphase comparative study for WHO/ISUP nuclear grading diagnostic model based on enhanced CT images of clear cell renal cell carcinoma

**DOI:** 10.1038/s41598-024-60921-x

**Published:** 2024-05-27

**Authors:** Chenyang Lu, Yangyang Xia, Jiamin Han, Wei Chen, Xu Qiao, Rui Gao, Xuewen Jiang

**Affiliations:** 1 School of Control Science and Engineering, Shandong University, Jinan, 250100 People’s Republic of China; 2 Key Laboratory of Urinary Precision Diagnosis and Treatment, Department of Urology, Qilu Hospital, Cheeloo College of Medicine, Shandong University, Jinan, 250012 People’s Republic of China; 3 Department of Radiology, Shandong First Medical University and Shandong Academy of Medical Sciences, Taian, People’s Republic of China

**Keywords:** Clear-cell renal cell carcinoma, WHO/ISUP nuclear grade, Enhanced computed tomography, Machine learning, Nephrology, Engineering

## Abstract

To compare and analyze the diagnostic value of different enhancement stages in distinguishing low and high nuclear grade clear cell renal cell carcinoma (ccRCC) based on enhanced computed tomography (CT) images by building machine learning classifiers. A total of 51 patients (Dateset1, including 41 low-grade and 10 high-grade) and 27 patients (Independent Dateset2, including 16 low-grade and 11 high-grade) with pathologically proven ccRCC were enrolled in this retrospective study. Radiomic features were extracted from the corticomedullary phase (CMP), nephrographic phase (NP), and excretory phase (EP) CT images, and selected using the recursive feature elimination cross-validation (RFECV) algorithm, the group differences were assessed using T-test and Mann–Whitney U test for continuous variables. The support vector machine (SVM), random forest (RF), XGBoost (XGB), VGG11, ResNet18, and GoogLeNet classifiers are established to distinguish low-grade and high-grade ccRCC. The classifiers based on CT images of NP (Dateset1, RF: AUC = 0.82 ± 0.05, ResNet18: AUC = 0.81 ± 0.02; Dateset2, XGB: AUC = 0.95 ± 0.02, ResNet18: AUC = 0.87 ± 0.07) obtained the best performance and robustness in distinguishing low-grade and high-grade ccRCC, while the EP-based classifier performance in poorer results. The CT images of enhanced phase NP had the best performance in diagnosing low and high nuclear grade ccRCC. Firstorder_Kurtosis and firstorder_90Percentile feature play a vital role in the classification task.

## Introduction

Renal cell carcinoma (RCC) accounts for more than 90% of renal cancers^1^, of which clear cell renal cell carcinoma (ccRCC) is the most common pathological type of RCC with poor prognosis, accounting for 70% of renal malignant tumors^2^. The standard-of-care treatment for ccRCC is closely related to the pathological grade^3,4^ and metastatic disease relies on systemic treatments. Surgery is still considered as the standard and curative option for localized ccRCC. However, approximately 30% of initially diagnosed localized ccRCC patients have high-risk features and the risk of recurrence is above 40%^5^. For these patients, the implementation of intensive management has been reported to improve overall survival, and pre-operative neoadjuvant treatments with target therapies or immunotherapies have been investigated to decrease the risk of recurrence in high-risk localized ccRCC. Therefore, it is vital to distinguish suitable patients with high-risk features at early stages and select the optimal strategy. For now, several models using different factors have been proposed to define the high-risk group^6^. Among these predictive factors, pathological nuclear grade is extensively studied and considered to be one of the most important prognostic factors for ccRCC. And patients with high nuclear grade are at greater risk of postoperative recurrence and poor prognosis. At present, the WHO/ISUP pathological nuclear grading system, as the mainstream grading standard of ccRCC, has been widely proven to accurately distinguish grading groups and predict different prognosis of RCC patients^7^. Although renal tumor biopsy (RTB) is regarded as the gold standard for assessing nuclear grading of ccRCC^8^, RTB is a redundant invasive procedure with unavoidable safety risks. Besides, the grade evaluation needs the histological evaluation. Generally speaking, small lesions should be completely sampled, and large lesions should adopt an effective sampling scheme, to get a sample well representative of the tumor heterogeneity is the challenge^9^. Therefore, the use of non-invasive methods to identify high-risk factors such as nuclear grading before surgery is of great significance for guiding clinical practice.

A multiphase contrast-enhanced CT scan of the kidney is an essential means of early imaging diagnosis of ccRCC, and studies have pointed out that the dynamic enhancement degree of ccRCC tumors on the image is a crucial indicator for the diagnosis of renal malignant tumors, which is related to the intensity uniformity of the tumor tissue intensity^10–12^. When distinguishing low nuclear grade from high nuclear grade ccRCC, the intensity and intensity uniformity of tumor tissues are different at different enhancement stages. Secondly, the renal structure, pathology, and urinary tract structure can be observed in different enhancement stages. In addition, there may be other features that have not been discovered artificially, so we choose radiation features from different enhancement stages to distinguish low-level nuclear ccRCC from high-level nuclear ccRCC. However, it is difficult for the doctor to observe the nuances of the intensity uniformity between different enhancement phases with the naked eye, which will greatly reduce their work efficiency. In recent years, radiomics has been an important means of non-invasive diagnosis of renal diseases. Based on abdominal CT and MRI, combined with machine learning technology, preoperative detection, typing, and grading of RCC can be achieved, which can largely avoid invasive testing risk^13–15^. There have been several image diagnostic studies on ccRCC to obtain features from contrast-enhanced CT and predict the Furman nuclear grade^16–18^, but only a few studies analyzed the WHO/ISUP grading system^19–21^. Whereas the WHO/ISUP nuclear grade system has been proven to have better prediction performance compared with the Fuhrman system in cancer-specific survival and overall survival^22^. To our knowledge, no studies have compared and analyzed the potential differences between different enhancement phases of CT images in the WHO/ISUP nuclear grade of ccRCC, and there is no consensus on which enhancement phase images should be used preferentially by doctors for diagnosis.

Therefore, the purpose of our study was to compare and evaluate the radiological features extracted from various enhancement epochs of ccRCC contrast-enhanced CT images, and to construct machine learning models to derive the best phase to diagnose the WHO/ISUP nuclear grading results.

## Materials and methods

**Figure 1:**
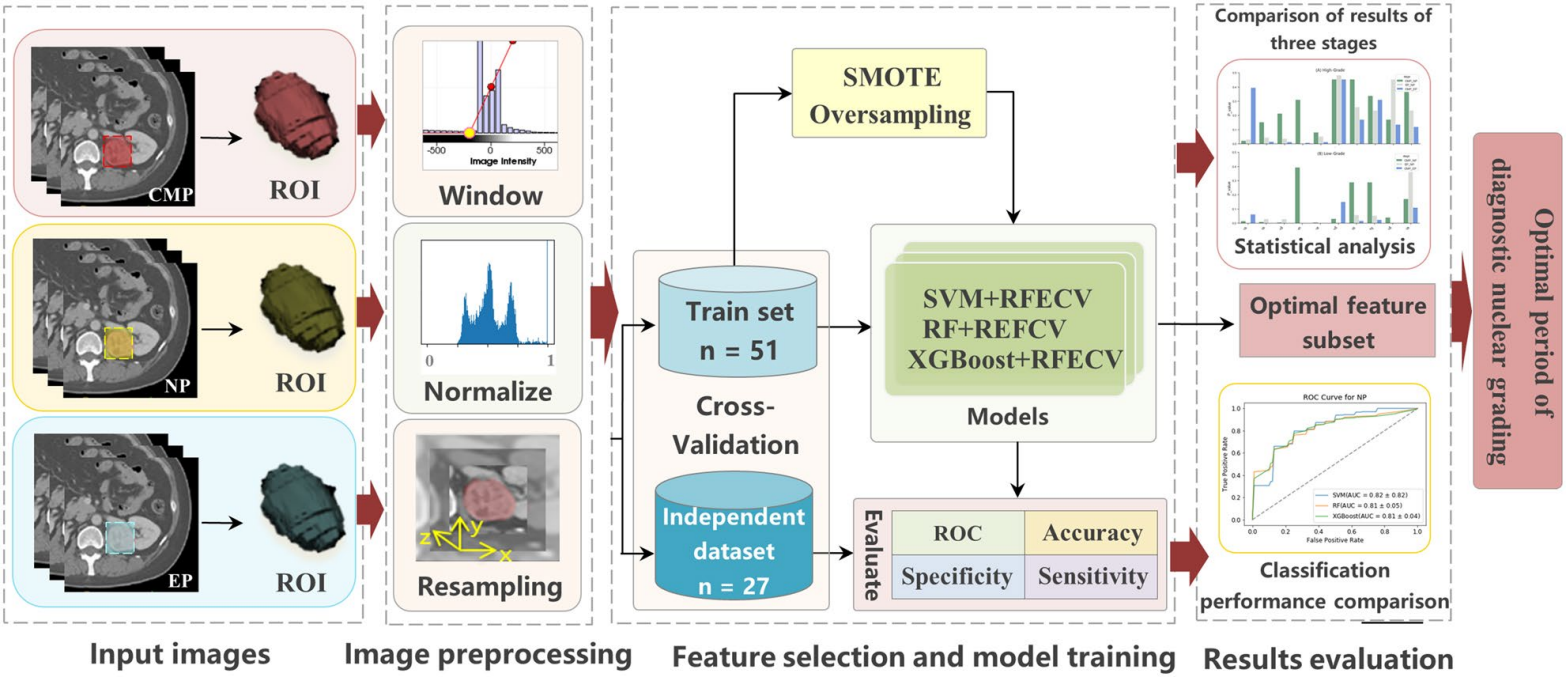
The technical route flow chart of the whole research.

The overall flow chart of the study is shown in Fig. 1. The details of each step will be introduced one by one later.

### Study patients

**Figure 2:**
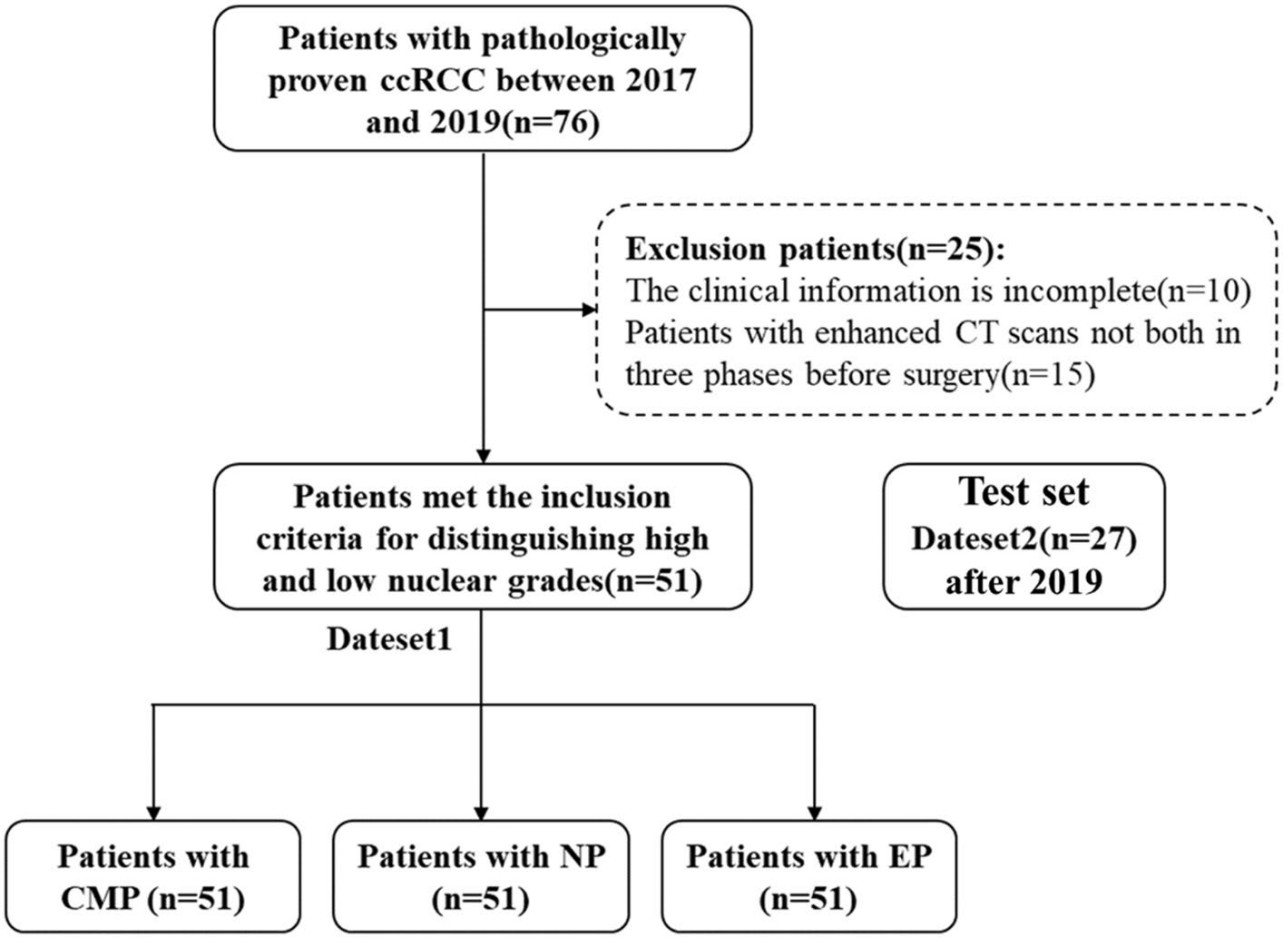
Flowchart of study patients.

This study was approved by the Institutional Ethics Committee of Qilu hospital (Num: KYLL-202205-035-1). Considering that enhanced CT examination is a routine noninvasive technique for suspected patients with ccRCC, the requirement for informed consent was waived. Patients with preoperative enhanced CT scanning and pathologically proven ccRCC between January 2017 and December 2019 were included in the study. The selection criteria for patients are as follows: (a) patients with ccRCC diagnosed by pathology; (b) patients with enhanced CT scans in three periods before surgery; (c) images and clinical data are complete. The patient recruitment flowchart is shown in Fig. 2. In the first four days of data set 1, 76 cases were collected, and 10 cases with incomplete clinical data and 15 cases with incomplete enhanced CT data in three periods were excluded. Finally, we included 51 case studies for training. In addition, we also used the same method to collect 27 cases of data after 2019 as an independent verification set.

### CT technique

We employed a SOMATOM Force CT scanner from Siemens Healthcare, located in Erlangen, Germany. Standard protocols were strictly adhered to for all patients. In brief, patients received 60–80 ml of non-ionic contrast agent, iopromide, administered intravenously into the forearm at a rate of 3–5 ml/s using a high-pressure syringe. The scanning protocol included the following phases: Corticomedullary Phase (CMP): The scanning delay time for this phase was set at 20–25 s. During this phase, contrast enhancement within the renal cortex and medulla is maximized, providing detailed imaging of the renal vasculature. Nephrographic Phase (NP): The scanning delay time for this phase was set at 50–60 s. This phase captures the peak enhancement of the renal parenchyma, allowing for visualization of renal structures and pathology. Excretory Phase (EP): Excretory phase scanning was performed 3–5 min after contrast injection. During this phase, contrast material is excreted into the renal collecting system and urinary tract, facilitating the visualization of urinary structures and evaluating for any excretory abnormalities.

### Histopathological assessment of nuclear grade

The WHO/ISUP nuclear grades were collected from the histopathological reports at Qilu Hospital of Shandong University. Histopathological evaluations were performed by two genitourinary pathologists with over 10 years of experience in the WHO/ISUP grading system. These grades are determined according to the cytological and nuclear morphological characteristics of renal cell carcinoma. Usually, after histological examination and microscopic observation, the tissue samples of renal cancer are judged according to the cell atypia (cell size, shape, and nuclear characteristics) and the structural characteristics of the tumor (such as the arrangement of tubular structures). Through microscopic observation and histological analysis of tumor tissue, the morphological characteristics of different parts of cells are determined, and the highest grade observed is our label grade. Even so, we think there may still be some deviations, so we divide grades 1 and 2 into low grades and grades 3 and 4 into high grades to form our later experimental data. In addition, because our research only focuses on the best time to distinguish between high-grade and low-grade nuclei, this experimental setup can effectively find the answer, so we designed the whole experiment.

### Image preprocessing

#### Resampling

In this experiment, because different patients may be photographed by different devices, the metadata of some CT images may be different, which may lead to incorrect feature extraction in the later stage. In order to avoid this problem, we first look at the metadata of CT images and find that the pixel spacing of CT images is slightly different. In order to unify the difference in layer thickness and in-plane resolution between images, the B-spline interpolation algorithm is used to uniformly sample all image data to an isotropic voxel size of 1 × 1 × 1 cm. The cubic B-spline interpolation algorithm we adopt is as follows: The pixel value I at the point $({x}{\prime},{y}{\prime})$ is obtained by using the pixel values of 4 × 4 integer points around it,

1 $$\begin{array}{c}I\left({x}{\prime},{y}{\prime}\right)=\sum\limits_{i=0}^{3} \sum\limits_{j=0}^{3} I\left(i,j\right)*W\left(i,j\right)\end{array}$$

where $W$ is the weight, and its expression is as follows

2 $$\begin{array}{c}W\left(i,j\right)=w\left({d}_{i}\right)*w\left({d}_{j}\right)\end{array}$$

3 $$\begin{array}{c}{d}_{i}=p\left(i,j\right)\cdot x-{x}{\prime}\end{array}$$

4 $$\begin{array}{c}{d}_{j}=p\left(i,j\right)\cdot y-{y}{\prime}\end{array}$$

5 $$\begin{array}{c}w(d)=\left\{\begin{array}{l}(a+2{)}^{*}\mid d{\mid }^{3}-(a+3{)}^{*}\mid d{\mid }^{2}+1\hspace{0.25em}\hspace{0.25em}\hspace{0.25em}\hspace{0.25em},\mid d\mid \le 1\\ {a}^{*}\mid d{\mid }^{3}-{5}^{*}{a}^{*}\mid d{\mid }^{2}+{8}^{*}{a}^{*}\mid d\mid -{4}^{*}a\hspace{0.25em}\hspace{0.25em}\hspace{0.25em}\hspace{0.25em},1<\mid d\mid \le 2\\ 0\hspace{0.25em}\hspace{0.25em}\hspace{0.25em}\hspace{0.25em},else\end{array}\right.\end{array}$$

where the value range of $a$ is − 1 to 0, generally a fixed value is − 0.5, and $p(i,j)\cdot x$ and $p(i,j)\cdot y$ respectively represent the $x$ coordinate and $y$ coordinate of the point $p(i,j)$, and $d$ is an intermediate substitution value. The weight $w$ returns to update our initial pixel value to realize cubic B-spline interpolation algorithm.

#### Window width and level adjustment

Different human tissues or lesions have different CT values. If we want to observe a certain range of tissues, we should choose the appropriate window width and window level, and modifying the window width and window level is usually called the adjustment of contrast brightness. Therefore, improper window width and window level will lead to image blurring and interfere with our feature extraction and image marking of the kidney, so it is necessary to adjust window width and window level for CT images. In this experiment, according to the experience and the gray value of the image in the region of interest, we set the window level to 0 Hu and the window width to 400 Hu by using the SetWindow and SetOutput methods in the IntensityWindowingImageFilter package in SimpleITK.

#### Artifact correction and image denoising

Especially for images with poor imaging effects, unified artifact correction and image denoising are needed to avoid the extracted features containing a lot of noise. In this experiment, based on the histogram of the image, the distribution difference of pixel values in the image is used to detect the artifact region. In addition, the voxel values are discretized to reduce image noise and improve the calculation efficiency of imaging features.

#### Pixel value standardization

In addition, in order to speed up the convergence speed of model training, this experiment standardized the image pixel value to make its numerical range between 0 and 1. The formula is as follows:

6 $$\begin{array}{c}norm=\frac{{x}_{i}-\mathit{min}\left(x\right)}{\mathit{max}\left(x\right)-\mathit{min}\left(x\right)}\end{array}$$

where ${x}_{i}$ represents the value of the image pixel, $\mathit{min}\left(x\right)$ and $\mathit{max}\left(x\right)$ represent the minimum and maximum values of the image pixel respectively.

#### Feature normalization

In addition, for the image genomics model, after extracting the image features, the features are also a set of data composed of numbers, similar to image pixels. Therefore, in order to unify the feature dimension and improve the training efficiency of the model, the experiment normalized the features. The processing method is the same as the pixel value standardization method.

### Tumor segmentation and feature extraction

All CT images are exported from the Picture Archiving and Communication System (PACS) and then transferred to a separate workstation for manual segmentation using ITKSNAP 3.6 software (www.itk-snap.org). For each tumor, the urologist draws a free 3D region of interest (ROI) on each slice of three -phase enhanced CT. Texture feature extraction was accomplished (with Pyradiomics) on the delineated ROIs in each phase. The 100 candidate features were extracted from each phase based on the original filter, including 18 first-order features, 11 shape features (3D), 3 shape features (2D), 22 Gy level co-occurrence matrix (GLCM) features, 16 Gy level run-length matrix (GLRLM) features, 16 Gy level size zone matrix (GLSZM) features, and 14 Gy level dependence matrix (GLDM) features. See Supplementary Table 1 for the whole feature of extraction. Z-score normalization are used to eliminate the influence between different features.

### Data oversampling

From the point of view of the training model, if the number of samples in a certain category is small, then the information provided by this category is too small, and the model will be more inclined to a large number of categories, which is obviously not what we want to see. The high-grade and low-grade data distributions are not balanced in our study, there were 10 cases of low grade and 41 cases of high grade, which would directly result in the model being more capable of learning the majority class (low-grade) than the minority class (high-grade) when training the model. Synthetic minority oversampling technique (SMOTE) is used by us to solve this problem, which changes the data distribution of an imbalanced dataset by adding generated minority class samples, and is one of the popular methods to improve the performance of classification models on imbalanced data^23^. First, each sample $Xi$ is sequentially selected from the minority class samples as the root sample for synthesizing new samples; secondly, according to the up-sampling magnification $n$, a sample is randomly selected from $k$ ($k$ is generally an odd number, such as $k=5$) neighboring samples of the same category of $Xi$ as an auxiliary sample for synthesizing a new sample, and repeated $n$ times; then linear interpolation is performed between sample $Xi$ and each auxiliary sample by the following formula, and finally $n$ synthetic samples are generated.

7 $$\begin{array}{c}{x}_{\text{new,attr}}={x}_{i\text{,attr}}+\left({x}_{ij\text{,attr}}-{x}_{i\text{,attr}}\right)\times \gamma \end{array}$$

where ${\varvec{x}}_{i}  \in {{\textbf{R}}}^{d}$, ${x}_{i\text{,attr}}$ is the $attr$-th attribute value of the $i$ -th sample in the minority class, $attr={1,2},\ldots , d$; $\gamma$ is a random number between^1^; $Xij$ is the $j$-th neighbor sample of sample $Xi$, $j={1,2},\ldots ,k$; ${x}_{new}$ represents a new sample synthesized between $Xij$ and $Xi$. The characteristic dimension of our sample is 2-dimensional, so each sample can be represented by a point on the 2-dimensional plane. A new sample ${x}_{\text{new,attr}}$ synthesized by the SMOTE algorithm is equivalent to a point on the connecting line between the point representing the sample ${x}_{i\text{,attr}}$ and the point representing the sample ${x}_{ij\text{,attr}}$. We implemented SMOTE using Python's sklearn library. Since our dataset is small, the setting of random seeds may cause large differences in oversampling and training results. To address this instability, we chose 30 random The number of seeds, between 0 and 300, and the average result is taken at the end.

### Feature selection and model training

We use the wrapping RFECV feature selection method. The feature selection realized by the RFECV method is divided into two parts: the first is the RFE part, that is, recursive feature elimination, which is used to rank the importance of features; the second is the CV part, that is, cross-validation. After feature rating, the best number of features are selected through cross-validation. The specific process we use in this experiment is as follows:

#### RFE stage

(1) The initial feature set includes all available features. (2) Use the current feature set to model, and then calculate the importance of each feature. (3) Delete the least important feature(s) and update the feature set. (4) Skip to step 2 until the importance rating of all features is completed.

#### CV stage

(1) According to the importance of features determined in the RFE stage, different numbers of features are selected in turn. (2) Cross-check the selected feature set. (3) Determine the number of features with the highest average score and complete feature selection.

By building SVM^24^, RF^25^, and XGB^26^ classifiers, using five-fold cross-validation to train the model and perform grid parameter optimization in each fold training set, using SMOTE oversampling. The feature weight coefficient of liner SVM and the feature Gini coefficient of RF and XGB are used as the basis for RFECV to filter the feature set. Then the results are averaged by training 30 times with 30 oversampled random seeds, and the average is used to evaluate model performance.

#### Support vector machine

Given a data set $D=\{({\textbf{x}}_{1},{{\text{y}}}_{1}),({\textbf{x}}_{2},{{\text{y}}}_{2}),\ldots ,({\textbf{x}}_{{\text{n}}},{{\text{y}}}_{{\text{n}}})\}$, where ${\textbf{x}}_{i}\in {\mathbb{R}}^{m}$ represents the $m$-dimensional feature data, ${y}_{{\text{i}}}\in \{-1,+1\}$ represents the classification label, and $n$ is the total number of samples. Then the optimal hyperplane can be expressed as:

8 $$\begin{array}{c}{{\varvec{W}}}^{{\text{T}}}x+b=0\end{array}$$

where the column vector ${\varvec{W}}={({W}_{1},{W}_{2},\dots ,{W}_{m})}^{T}$ is the normal vector, which determines the direction of the hyperplane; $b$ is the displacement term, which together with the normal vector W determines the distance between the hyperplane and the origin. In order to maximize the classification interval, the objective function is:

9 $$\begin{array}{c}\underset{{\varvec{W}},b}{{\text{min}}} 0.5\parallel W{\parallel }^{2}+C\sum\limits_{i=1}^{n}{\xi }_{i}\end{array}$$

10 $$\begin{array}{c}{\text{s}}\text{.}{\text{t}}\text{.}{ y}_{i}\left({{\varvec{W}}}^{{\text{T}}}{{\varvec{x}}}_{i}+b\right)\ge 1-{\xi }_{i},i={1,2},\ldots ,n\end{array}$$

where $C>0$ represents the penalty factor, and the larger $C$ means the greater penalty for misclassification.

#### Random forest

When classifying, the RF classifier ${h}_{i}$ predicts a label from the set $\{c,{c}_{2},\dots ,{c}_{n-1},{c}_{n}\}$ that has been labeled, and expresses the predicted output of ${h}_{i}$ on the sample $x$ as an N-dimensional vector $({h}_{i}^{1}(x);{h}_{i}^{2}(x);\dots {h}_{i}^{N}(x))$*,* where ${h}_{i}^{j}{\text{x}}$ is the output of ${h}_{i}$ on the category label ${c}_{j}$, the voting process of RF is publicized as:

11 $$\begin{array}{c}H\left(x\right)={\text{arg}}{max}_{C}\left(\frac{1}{{n}_{\text{tree}}}\sum\limits_{i=1}^{{n}_{\text{tree}}}I\left(\frac{{n}_{hi}, c}{{n}_{hi}}\right)\right)\end{array}$$

In the formula, ${n}_{\text{tree}}$ is the number of trees in the RF classifier; $I(*)$ is an indicative function; $({n}_{hi},c)$ is the classification output of the tree for type ${c}_{j}$; ${n}_{hi}$ is the number of leaf nodes of the tree. Therefore, RF is a highly generalized integrated learning model.

#### XGBoost

Assuming that there is a data set containing independent variables $X=\{{x}_{1},{x}_{2},\dots ,{x}_{m}\}$, classification variables ${y}_{i}$ and $n$ samples, $K$ CART trees are obtained by training them, and the final predicted value $\hat{{y}_{i}}$ is the accumulation of these tree models value:

12 $$ \begin{array}{*{20}c}    {\hat{y}_{i}  = \sum\limits_{{k = 1}}^{K} {f_{k} } \left( {x_{i} } \right) = \sum\limits_{{k = 1}}^{K} {w_{{kq_{k} \left( x \right)}} } }  \\   \end{array}  $$

In the formula, $f(x)$ represents a CART regression tree, ${w}_{k}$ is the leaf weight of the $k$-th regression tree, $q(x)$ is the number of the leaf node, that is, the sample $x$ will finally fall on a certain leaf node of the tree, its value is ${w}_{q(x)}$. XGB adds a regular term $\Omega (f)$ to the traditional loss function, and uses incremental learning to optimize the function, that is, starting from a constant, each round of training adds a new function on the basis of keeping the previous round of model unchanged, such as the predicted value ${\hat{y}}_{i}^{(t)}={\hat{y}}_{i}^{(t-1)}+{f}_{t}({x}_{i})$ of the $t$ -th round, then its loss function $L$ is:

13 $${L}^{\left(t\right)}=\sum_{i=1}^{n}l({y}_{i},{\hat{y}}_{i}^{(t-1)}+{f}_{t}({x}_{i}))+\Omega ({f}_{t})$$

where $\Omega (f)=\gamma T+{2}^{-1}\lambda \sum_{j=1}^{T}{w}_{j}^{2}$, $\gamma$ and $\uplambda$ are the parameters of the regular term, and $T$ represents the number of leaf nodes. XGB can improve the classification accuracy after combining many weak classifiers to form a powerful combined classifier.

### Statistical analysis

All statistical analyses were performed using Python (version 3.6.5). The group differences were assessed using a Mann–Whitney U test for continuous variables. The Mann–Whitney U test can be regarded as a substitute for the independent two-sample T test or the corresponding large-sample normal test for the parameter test of the difference between two means. Because the Mann–Whitney U test explicitly considers the rank of each measured value in each sample, it uses more information than a symbolic test. The original hypothesis that $H0$ is the distribution of two populations from which two independent samples come has no significant difference. If the probability $p$ value is less than or equal to a given significance level $\alpha$, the original hypothesis is rejected and the distribution of two populations from which samples come is considered to be significantly different. On the other hand, the original hypothesis is accepted, and there is no significant difference in the distribution of the two populations from which the samples come^27,28^. In this experiment, a $p$ value less than 0.05 indicates a statistically significant difference.

In the classification task, the prediction situation is divided into true positive (TP), true negative (TN), false positive (FP), and false negative (FN). Here, TP denotes the number of cases correctly predicted as positive, FP denotes the number of cases wrongly predicted as positive, TN denotes the number of cases correctly predicted as negative, and FN denotes the number of cases wrongly predicted as negative. The aforementioned four cases can be expressed using a confusion matrix to evaluate the classification performance of the model. Based on the confusion matrix, the proportion of all correct prediction results in the total sample can be calculated using accuracy (ACC) as an evaluation indicator. The higher the index is, the more accurate the model prediction is, and the fewer samples are wrongly predicted.

14 $$\begin{array}{c}ACC=\frac{TP+TN}{TP+FN+FP+TN}\end{array}$$

Specificity indicates the sensitivity to negative samples. Essentially, the higher the accuracy rate, the lower the false detection rate.

15 $$\begin{array}{c}SPE=\frac{TN}{TN+FP}\end{array}$$

Sensitivity, also known as true positive rate, Recall rate, indicates the proportion of correctly predicted positive samples to actual positive samples, and the higher the sensitivity, the lower the missed detection rate.

16 $$\begin{array}{c}SEN=\frac{TP}{TP+FN}\end{array}$$

F1 value (F1-measure) comprehensively indicates the accuracy and sensitivity results. The more proximal the index is to one, the more satisfactory the output result of the model.

17 $$\begin{array}{c}F1=\frac{2\times TPR\times PPV}{TPR+PPV}\end{array}$$

The receiver operating characteristic (ROC) curve based on sensitivity and specificity plots TPR on the y-axis and 1 − TNR on the x-axis. The area under the curve (AUC) value, representing the area under the ROC curve, is utilized to evaluate the classification ability of the model. A higher AUC value signifies superior model performance and enhanced classification ability. Finally, through the analysis of the results, we can get the best period of diagnostic nuclear grading.

### Ethical approval

The study involving human subjects was reviewed and approved by the Ethics Committee of the Qilu Hospital (No: KYLL-202205-035-1).

### Consent statement

All methods were performed in accordance with the relevant guidelines and regulations. All participants gave their written informed consent.

## Results

### General characteristics

The training set for this study included 41 patients in the low-grade group and 10 patients in the high-grade group, and the baseline characteristics are shown in Table 1. It contains eight clinical features: Age (years), Gender, Weight (kg), Height (CM), BMI, Tumor Side (left and right), Tumor Location (upper pole, middle, and lower pole) and Tumor Size (cm). The significant difference between the two groups of data was analyzed, and the results showed that there was no significant difference in age (p = 0.5141), gender (p = 0.3717), weight (p = 0.7291), height (p = 0.5678), and BMI between two groups. Moreover, the difference in tumor side (p = 0.5553) and tumor location (p = 0.4593) was not significant, but patients in the high-grade group have significantly larger tumor size compared with those in the low-grade group (p = 0.043 < 0.05). It shows that the size of the tumor has a significant influence on the distinction between high-grade and low-grade groups in the clinic.

**Table 1:** Clinical characteristics of patients in low-grade and high-grade groups.

Characteristics	Low grade (n = 41)	High grade (n = 10)	P-value
Age (years)	57 (31–79)	64 (53–80)	0.5141
Gender			0.3717
Male	26	9	
Female	15	1	
Weight (kg)	71.65	71.83	0.7291
Height (CM)	168.27	168.13	0.5678
BMI	25.6	25.36	
Tumor side			0.5553
Left	19	3	
Right	22	7	
Tumor Location			0.4593
Upper pole	14	4	
Middle	19	4	
Lower pole	8	2	
Tumor Size (cm)	3.62	4.81	0.043

### Feature extraction and selection

**Figure 3:**
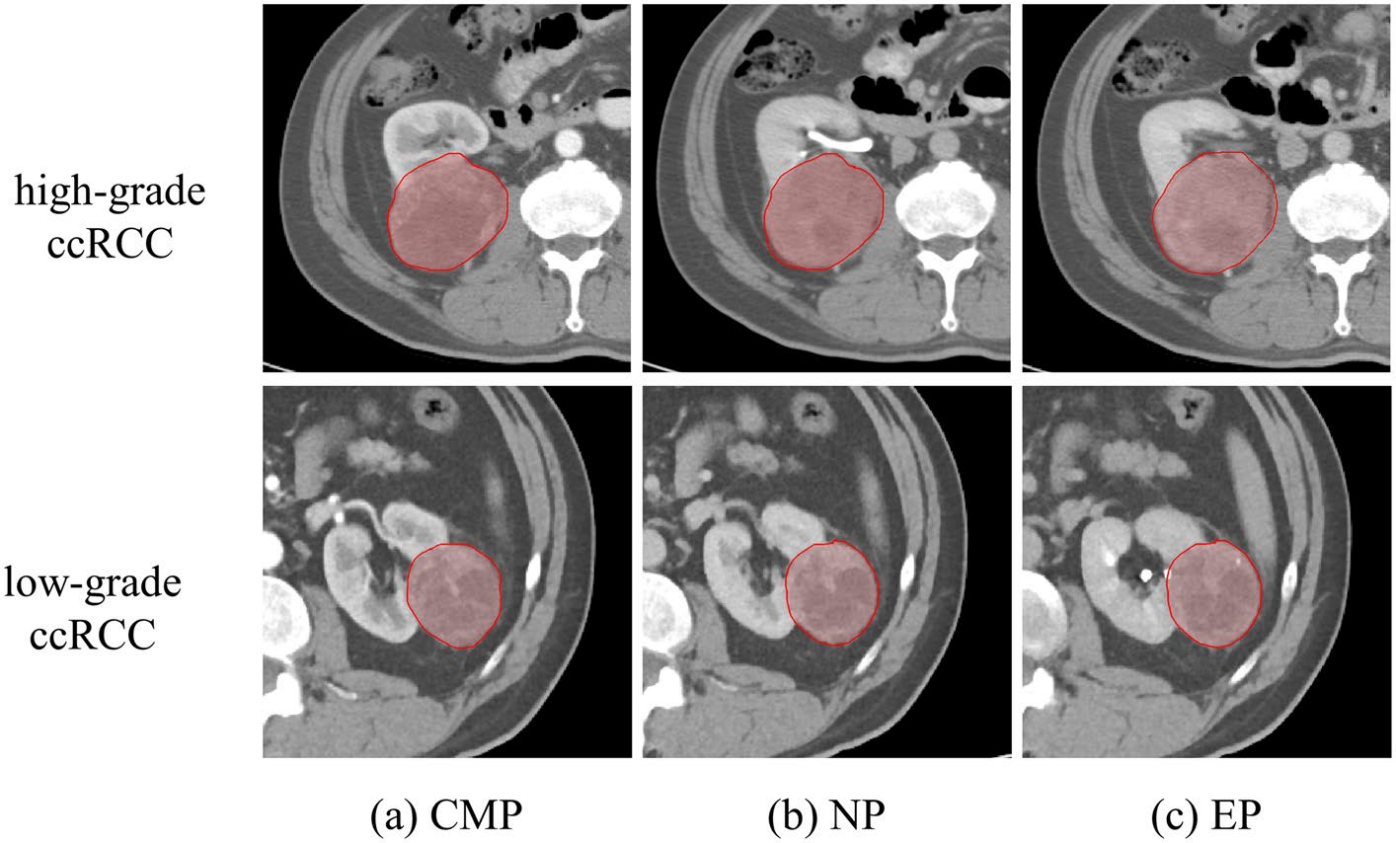
An example of the manual segmentation on multiphase CT of high-grade and low-grade ccRCC. (**a**–**c**) show the tumor regions of CMP, NP, and EP. Everyone also shows the ROI regions drawn based on the CT images.

Texture features are extracted from the region of interest (ROI) of multi-phase CT images, the results of manual tumor segmentation at different periods are shown in Fig. 3. In total, we extracted 100 radiomics features from each phase. In each stage, three classifiers, SVM, RF, and XGBoost, are used for feature selection in combination with RFECV. The model is trained 30 times according to the 30 random seeds selected by SMOTE oversampling, and a feature subset is obtained by training RFECV each time. The number of occurrences of each feature in the 30 feature subsets is selected, and the features that appear no less than 15 times are chosen for analysis. In dataset1 and dataset2, the features selected using the three classifiers for the CMP, EP, and NP epochs, respectively, are shown in Supplementary Tables 2–7. In dataset1, 19, 4, and 3 features were selected by SVM, RF, and XGB classifiers in NP, respectively. Moreover, 4, 15, and 3 features were selected in CMP, respectively, and 8, 8, and 3 features were chosen in EP. Which will further verify the robustness and reproducibility of the selected features.

### Model training and results analysis

**Figure 4:**
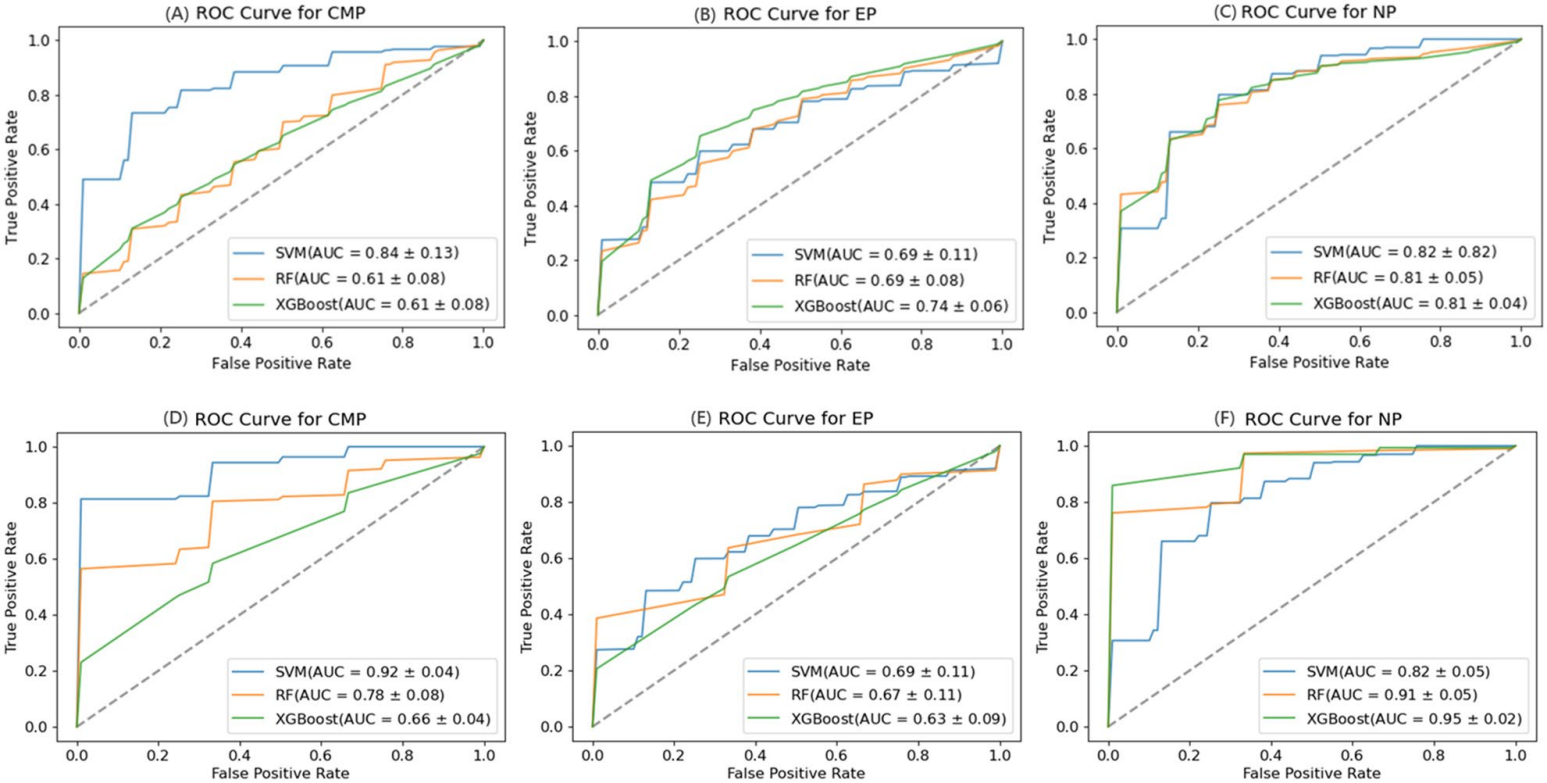
ROC curves of the SVM, RF, and XGB classifiers based on the CT image of CM,EP, and NP in Dateset1 and Dateset2: (**A**–**C**) show the ROC covers based on CMP,EP, and NP in Dateset1; (**D**–**F**) show the ROC covers based on CMP、EP and NP in Dateset2.

In this study, we constructed three classifiers, SVM, Random Forest, and XGBoost for the three stages of CMP, NP, and EP, respectively, using the feature subset selected by RFECV to input the model for training, and using five-fold cross-validation for model validation and evaluate. According to the average of 30 results of training with 30 random seed numbers, the ROC curve of the model for predicting WHO/ISUP nuclear grade by CT image features of three stages is obtained as shown in Fig. 4. In dataset1 and dataset2, the average accuracy, sensitivity, specificity, AUC and F1 score of the three classifiers are shown in Table 2. We find that the NP has better robustness in discriminating kernel grades, while all three classifiers exhibit good performance (Dateset1, SVM: AUC = 0.82 ± 0.05, RF: AUC = 0.82 ± 0.05, XGB: AUC = 0.81 ± 0.04; Dateset2, SVM: AUC = 0.84 ± 0.14, RF: AUC = 0.91 ± 0.05, XGB: AUC = 0.95 ± 0.02).

**Table 2:** The machine learning models on the CT image features of CMP、EP and NP in Dataset1 and Dataset2. The best results in the table are marked in bold, and the second-best results are underlined.

Stage	Classifiers	AUC	ACC	SPE	SEN	F1
Dataset1						
CMP	SVM	**0.84 ± 0.13**	0.76 ± 0.10	0.77 ± 0.11	**0.70 ± 0.14**	**0.52 ± 0.14**
	RF	0.61 ± 0.08	0.69 ± 0.04	0.81 ± 0.05	0.21 ± 0.13	0.17 ± 0.10
	XGB	0.61 ± 0.08	0.56 ± 0.10	0.57 ± 0.15	0.53 ± 0.15	0.3 ± 0.09
NP	SVM	0.82 ± 0.05	**0.80 ± 0.04**	**0.87 ± 0.03**	0.50 ± 0.12	0.43 ± 0.10
	RF	0.82 ± 0.05	0.78 ± 0.03	0.85 ± 0.05	0.51 ± 0.09	0.45 ± 0.08
	XGB	0.81 ± 0.04	0.70 ± 0.08	0.71 ± 0.11	0.68 ± 0.17	0.47 ± 0.12
EP	SVM	0.69 ± 0.11	0.69 ± 0.11	0.72 ± 0.15	0.54 ± 0.13	0.41 ± 0.09
	RF	0.69 ± 0.08	0.68 ± 0.06	0.73 ± 0.08	0.46 ± 0.16	0.33 ± 0.11
	XGB	0.74 ± 0.06	0.66 ± 0.09	0.67 ± 0.13	0.60 ± 0.12	0.38 ± 0.06
Dataset2 (Independent data)						
CMP	SVM	0.92 ± 0.04	**0.82 ± 0.06**	**0.86 ± 0.06**	0.77 ± 0.09	**0.77 ± 0.07**
	RF	0.78 ± 0.08	0.60 ± 0.08	0.57 ± 0.10	0.69 ± 0.08	0.55 ± 0.10
	XGB	0.66 ± 0.04	0.58 ± 0.05	0.53 ± 0.12	0.67 ± 0.10	0.54 ± 0.06
NP	SVM	0.84 ± 0.14	0.73 ± 0.12	0.71 ± 0.13	**0.79 ± 0.14**	0.69 ± 0.14
	RF	0.91 ± 0.05	0.71 ± 0.11	0.71 ± 0.14	0.74 ± 0.13	0.65 ± 0.13
	XGB	**0.95 ± 0.02**	0.72 ± 0.10	0.72 ± 0.12	0.74 ± 0.14	0.65 ± 0.13
EP	SVM	0.83 ± 0.00	0.74 ± 0.02	0.75 ± 0.00	0.75 ± 0.06	0.69 ± 0.04
	RF	0.67 ± 0.11	0.55 ± 0.10	0.55 ± 0.17	0.58 ± 0.09	0.47 ± 0.08
	XGB	0.63 ± 0.09	0.60 ± 0.07	0.70 ± 0.10	0.43 ± 0.07	0.35 ± 0.10

**Figure 5:**
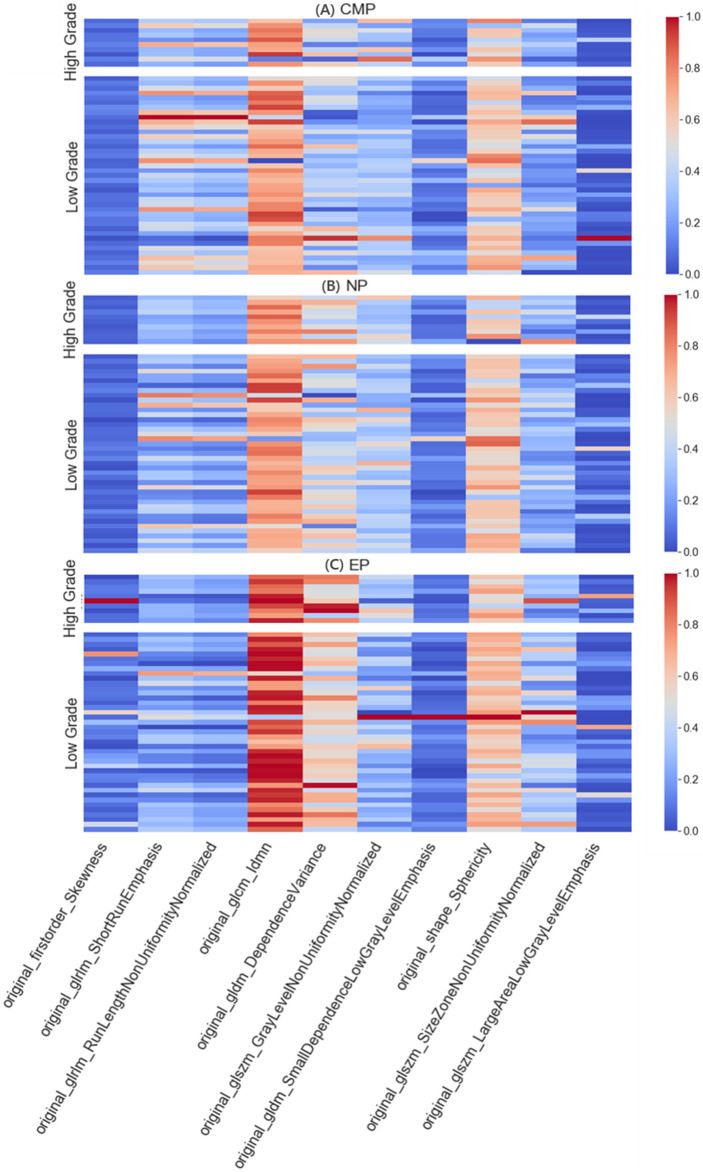
The heat map shows the distribution of normalised texture feature values between low and high nuclear grade ccRCC based on CMP, NP, and EP. Difference in colors and their shades indicates dissimilarity of the corresponding texture value parameters.

**Figure 6:**
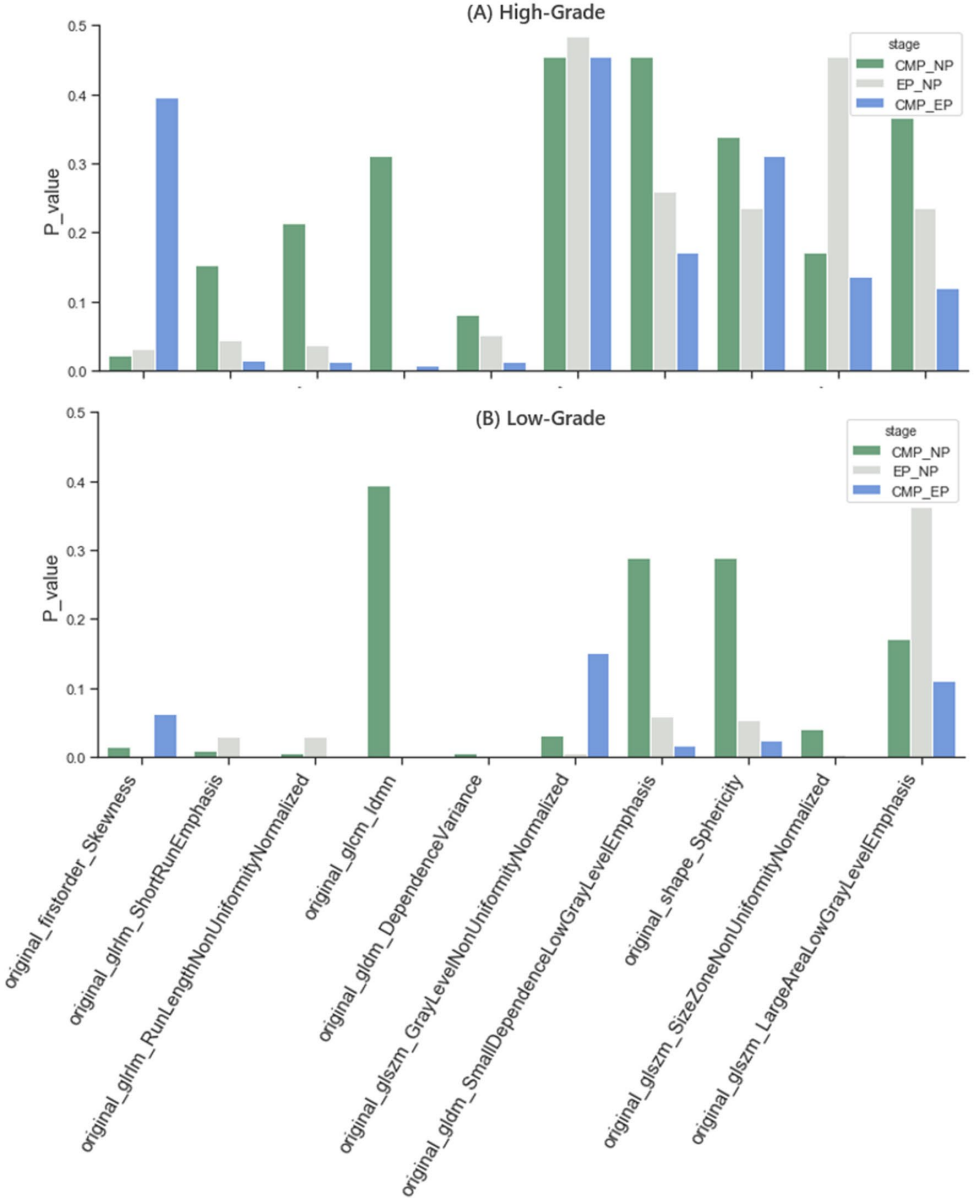
Distribution map of the difference p-values of characteristics in the two-point period: (**A**) high-level group; (**B**) low-level group.

We select 10 features that appear more than twice in the feature subsets of dataset1 selected by the three classifiers respectively in each stage, normalize them, and draw a heat map of the distribution of high and low kernel hierarchical features, the results are shown in Fig. 5. Among them, at each stage, three features, glcm_Idmn, gldm_DependenceVariance, and shape_Sphericity, all appear frequently, which fully demonstrates the importance of these three features. Compared with these features, the order of importance of firstorder_Skewness, glszm_LargeAreaLowGrayLevelEmphasis, and other features is relatively backward. The Mann Whitney U test was performed on the 10 features of the three stages, and the pairwise differences between the three stages in high-grade and low-grade groups were drawn as p-value histograms respectively, as shown in Fig. 6. The p-value less than 0.05 was considered to be different between the three stages. Among them, it is worth noting that although feature glcm_Idmn has a high frequency in other stages, it has a very high frequency in the EP stage, which is obviously different from other stages. In particular, the performance of the gldm_DependenceVariance feature is not obvious in other stages, but it has a relatively obvious high frequency in the NP stage, showing differences. Other features with high frequency and significant differences between CMP and other stages are mostly shape and texture features, which are consistent with the previous clinical characteristics analysis.

Specifically, in our study, we found that 4/5 of the selected important features were texture features, except for one first-order feature and another shape feature, while the overall discrepancy of features among the three stages in the low nuclear grade group was more significant than in the high nuclear grade group (mean p-value less than 0.08), which indicating that CT image texture in low-grade tumor had greater differences in three enhancement stages. In addition, the first-order feature Skewness ranked forward in the assessment of feature importance during NP and showed a significant difference compared with the other two stages (p < 0.05), which indicated that the CT image intensity fluctuation of tumors is the largest during NP and the lesion area is easier to detect on it. The value of the feature glcm_Idmn in EP is significantly larger than that in CMP (p < 0.01) and NP (p < 0.01), which mainly measures the local uniformity of the image. This indicates that the contrast enhancement rate of ccRCC CT images in EP basically disappeared, resulting in poor performance in distinguishing nuclear grades. Additionally, altered cellular permeability, abnormal angiogenesis, and necrosis of malignant tumors cause structural changes in the tissue and resulting in tumor heterogeneity in NP and CMP. However, only one shape feature appeared in the selected important features, and it did not show a significant difference in the three stages regardless of whether it was in a high-grade group or a low-grade group, so we speculate that the value of tumor shape feature is approximately same in distinguishing nuclear grades in three stages.

**Figure 7:**
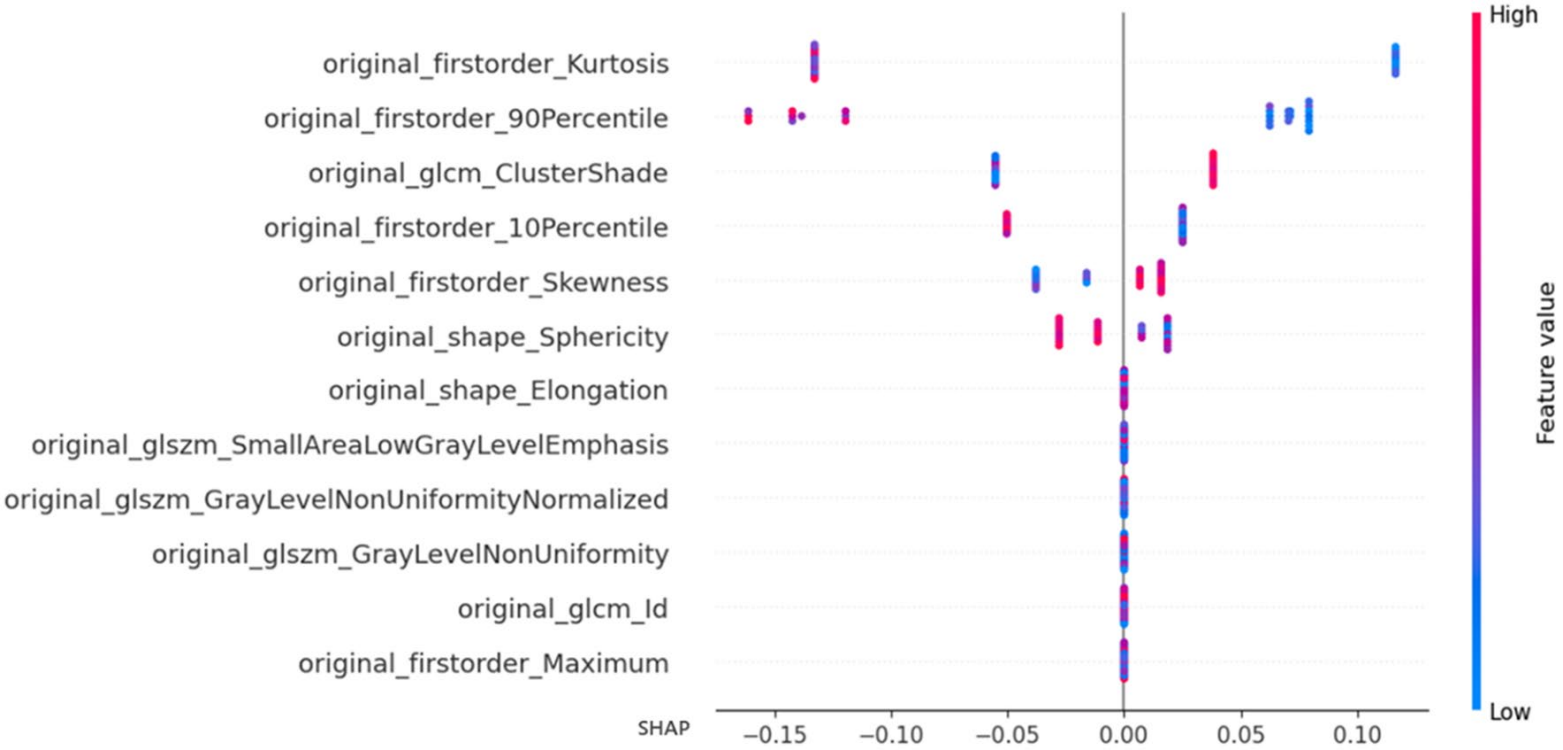
Visualization of shap value feature decision of XGBoost optimal model trained in dataset2.

For the XGBoost optimal model trained in dataset2, we visualize the feature decision of NP through the shape value as shown in Fig. 7. SHAP values are a method used to assess feature importance in machine learning models. They quantify the impact of each feature on model predictions. By understanding SHAP values, we can gain insights into the contribution of individual features and improve model interpretability. From SHAP values, the features firstorder_Kurtosis and firstorder_90Percentile exhibit the most significant impact on the model's predictive results, followed by the features glcm_ClusterShade, firstorder_10Percentile, firstorder_Skewness, and shape_Elongation. First-order features such as Kurtosis, 90Percentile, 10Percentile, and shape feature Elongation demonstrate an inverse relationship (i.e., when their values are high, they have a negative effect on the model's predictions, indicating a tendency to predict low-grade ccRCC; conversely, when their values are low, the model tends to predict high-grade ccRCC). This suggests that first-order features represent the grayscale distribution, with low-grade ccRCC exhibiting a more uniform grayscale value distribution and high-grade ccRCC showing the opposite pattern. Texture feature ClusterShade and first-order feature Skewness show a direct proportionality with the predictive results, indicating that higher-level tumor CT images exhibit a higher degree of pixel value aggregation within the local region, leading to more heterogeneous texture features in the image. This implies the presence of a wide range of pixel value aggregations in the image, possibly reflecting irregular or heterogeneous structures within the tissue, corresponding to complex texture features within the tumor region.

### Deep learning method

In order to further study the multi-phase comparison of WHO /ISUP nuclear grading diagnosis models based on enhanced CT images of renal clear cell carcinoma, we also designed an end-to-end nuclear grading classification model of deep learning, in which three end-to-end deep learning models, VGG11, ResNet18 and GoogLeNet, were constructed based on three enhancement periods: CMP, NP, and EP, and trained on the test set.

Like the radiomics methods, the deep learning model also needs to preprocess the input CT image data before training. In addition to adjusting the window width and level, resampling, and standardizing the gray value of the image as in the previous chapter, it is also necessary to design preprocessing steps such as image data enhancement and region of interest interception for the deep learning model to increase the diversity of training data and adapt to the input requirements of the model. In the experiment, the CT image data of the training set was enlarged and a few samples were balanced. Specifically, after each tumor area image was taken as a block, it was rotated by 90° and 180° respectively, and the training set was enlarged by mirroring it left and right and up and down. In order to meet the requirements of the deep learning model in this experiment for input images, a frame with the largest tumor area is selected, and a total of three layers of gray images are taken as the input of RGB three channels. After intercepting each image data, it needs to be scaled to a size of 224 × 224 px before entering the network.

Specifically, in the process of model training, the weight and bias of the model are randomly assigned by random initialization. The ratio of Dropout is set to 0.5, L2 regularization is introduced, and the coefficient is set to 0.5. The model takes the cross entropy loss function of classification problem as the optimization objective function, and the weight attenuation coefficient of network parameters is set to 0.0005. In order to reduce the shock in the process of model convergence and the randomness in training, Adam optimizer is used to optimize the objective function in the experiment. Set the size of Batch_size to 8. The initial learning rate is 1e − 4. CosineAnnealingLR method is used to decrease the learning rate. The model trains a total of 300 epochs, based on Pytorch deep learning framework, and uses NVIDIATeslaV100GP for training.

**Figure 8:**
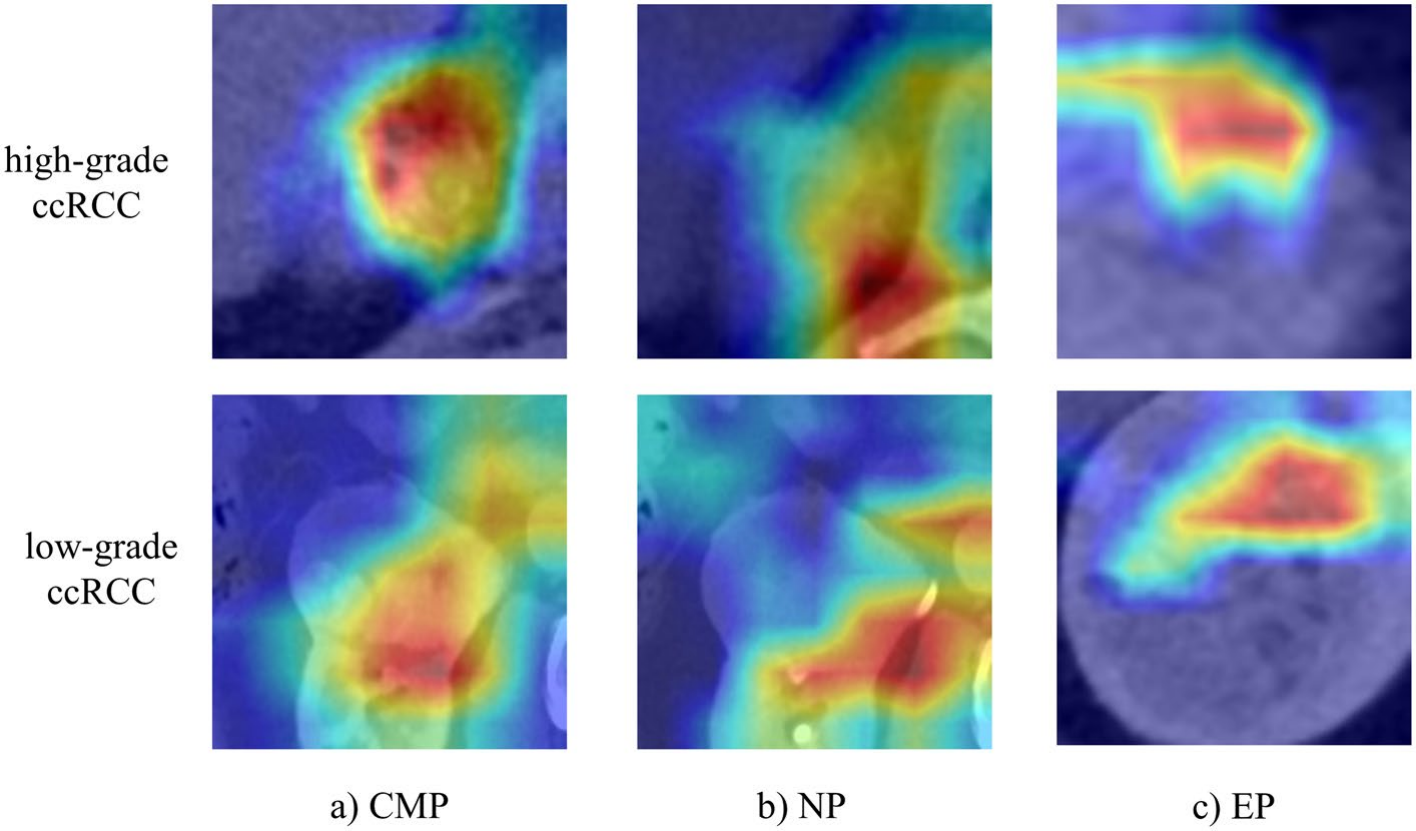
Visualization of attention areas for classification of ResNet18. The first line is a high-grade ccRCC, and the second line is a low-grade ccRCC. (**a**–**c**) show the tumor regions of CMP, NP, and EP.

Finally, in dataset1 and dataset2, the average accuracy, sensitivity, specificity, AUC and F1 score of the three classifiers are shown in Table 3. We also find that the NP has better robustness in discriminating kernel grades. Among them, ResNet18 shows the best performance (Dateset1, VGG11: AUC = 0.59 ± 0.04, ResNet18: AUC = 0.81 ± 0.02, GoogLeNet: AUC = 0.78 ± 0.04; Dateset2, VGG11: AUC = 0.68 ± 0.11, ResNet18: AUC = 0.87 ± 0.07, GoogLeNet: AUC = 0.57 ± 0.06). In addition, as shown in Fig. 8, we also draw the heat map of the region of interest of images in different periods when ResNet18 classifies high-grade and low-grade ccRCC.

**Table 3:** The deep learning models on the CT image features of CMP, EP and NP in Dataset1 and Dataset2. The best results in the table are marked in bold, and the second-best results are underlined.

Stage	Classifiers	AUC	ACC	SPE	SEN	F1
Dataset1						
CMP	VGG11	0.64 ± 0.10	0.70 ± 0.04	0.75 ± 0.14	**0.74 ± 0.02**	**0.60 ± 0.03**
	ResNet18	0.79 ± 0.04	0.70 ± 0.07	0.75 ± 0.09	0.63 ± 0.17	0.55 ± 0.11
	GoogLeNet	0.55 ± 0.07	0.56 ± 0.15	0.56 ± 0.05	0.55 ± 0.05	0.40 ± 0.08
NP	VGG11	0.59 ± 0.04	0.63 ± 0.09	0.75 ± 0.07	0.45 ± 0.13	0.51 ± 0.06
	ResNet18	**0.81 ± 0.02**	**0.72 ± 0.04**	**0.75 ± 0.04**	0.66 ± 0.08	0.58 ± 0.05
	GoogLeNet	0.78 ± 0.04	0.72 ± 0.06	0.75 ± 0.06	0.67 ± 0.04	0.58 ± 0.09
EP	VGG11	0.68 ± 0.13	0.63 ± 0.08	**0.75 ± 0.04**	0.45 ± 0.04	0.51 ± 0.14
	ResNet18	0.66 ± 0.12	0.56 ± 0.15	0.56 ± 0.10	0.55 ± 0.10	0.51 ± 0.13
	GoogLeNet	0.78 ± 0.08	0.72 ± 0.09	0.75 ± 0.13	0.67 ± 0.06	0.58 ± 0.10
Dataset2 (Independent data)						
CMP	VGG11	0.66 ± 0.10	0.67 ± 0.14	0.63 ± 0.08	0.73 ± 0.08	0.69 ± 0.15
	ResNet18	0.79 ± 0.03	0.70 ± 0.07	0.75 ± 0.05	0.64 ± 0.13	0.80 ± 0.09
	GoogLeNet	0.54 ± 0.09	0.56 ± 0.09	0.67 ± 0.15	0.33 ± 0.07	0.67 ± 0.03
NP	VGG11	0.68 ± 0.11	0.63 ± 0.14	0.63 ± 0.12	0.64 ± 0.17	0.67 ± 0.11
	ResNet18	**0.87 ± 0.07**	**0.81 ± 0.05**	0.75 ± 0.08	**0.91 ± 0.09**	**0.83 ± 0.11**
	GoogLeNet	0.57 ± 0.06	0.61 ± 0.13	**0.75 ± 0.04**	0.33 ± 0.13	0.72 ± 0.08
EP	VGG11	0.68 ± 0.13	0.63 ± 0.07	0.75 ± 0.06	0.46 ± 0.04	0.71 ± 006
	ResNet18	0.72 ± 0.12	0.70 ± 0.04	0.75 ± 0.15	0.64 ± 0.05	0.75 ± 0.02
	GoogLeNet	0.57 ± 0.17	0.61 ± 0.12	0.75 ± 0.11	0.33 ± 0.14	0.72 ± 0.05

From the medical point of view, this enhancement mode in different periods is determined by the pathological characteristics of ccRCC. At CMP stage, ccRCC tumor began to get blood supply from renal parenchyma. Because of the high metabolic activity of ccRCC tumor cells, they would absorb more contrast media, which led to the gradual and significant enhancement of the tumor. However, in NP stage, the contrast agent began to penetrate into renal pelvis and ureter, and ccRCC reached a stable peak enhancement at this time, that is, the absorption of contrast agent in tumor tissue reached the highest point, which led to the most obvious contrast enhancement performance in tumor area on CT images. Therefore, this peak enhancement makes the characteristics of tumor more obvious on the CT images of NP, and in CMP stage, some CT images are close to the peak enhancement of NP, which may lead to the performance of some models similar to NP. Secondly, in EP stage, the contrast agent began to be excreted from the kidney, and the enhancement degree of ccRCC gradually weakened, so the tumor image characteristics were not significant, which led to the poor performance of the model.

## Discussion

Our research showed that NP was the optimal stage to diagnose WHO/ISUP nuclear grades, and the model performance was more robust. By comparing features extracted from CT images of different enhancement stages, and building machine learning models and deep learning models, we found that image features differed by enhancement stage at the same nuclear-grade level. Moreover, the enhancement intensity has the greatest difference between high-grade and low-grade in NP.

There is no consistent result from previous studies on which enhancement phase should be used to differentiate the ccRCC WHO/ISUP nuclear grades. Previous studies have assessed the textural features of chromophobe RCC and ccRCC on monophasic CT and predicted the potential discriminative role of Furhman nuclear grade^15,29,30^. Another study investigated the association between Furman nuclear-grade and CT images in three-phase ccRCC involving the plain scan period, CMP, and NP^31^. The result also confirmed that CT radiomic features can be considered as a useful and promising noninvasive methodology for preoperative evaluation Fuhrman grades in ccRCC. However, in recent years, the Furhman nuclear grading system has been controversial because of its poor interpretability and uncertain prognostic value^32–34^, and it was gradually replaced by the WHO/ISUP nuclear grading system, which was proposed by the International Urological Association and can accurately distinguish the nuclear grade of renal cancer patients^7^. The WHO/ISUP nuclear grading system was used in our study as the gold standard for differentiating nuclear grading, which has greater research value than previous studies. In addition, some studies have analyzed texture features and used them to differentiate WHO/ISUP nuclear grades. Nevertheless, they only included single-phase^19^, two-phase^20^, or three-phase^21,35,36^ CT, and no studies included images of the excretion period. To our knowledge, only one study included the EP and compared four-phase CT in predicting Furhman nuclear grade^37^. The results showed that image features extracted from the unenhanced phase CT images demonstrated a dominant classification performance, which was inconsistent with previous studies. Here, we performed a comprehensive comparative analysis of the three enhancement phases (including EP) and found that the model based on the nephrography period showed robust and excellent performance.

In a study on which phase should be used to differentiate ccRCC from other renal masses, the texture features of renal masses in three contrast enhancement phases were evaluated, including non-contrast enhancement [NECT], cortico-medulla [CM], and nephrogram [NG]. The results showed that the ccRCC varies in different enhancement stages, and the enhancement stage was an essential variable in the texture analysis of renal masses^38^. In addition, another texture analysis study on predicting Furhman nuclear grades of ccRCC discovered that after Laplace Gaussian filtering, there is a statistically significant difference between the entropy (fine) of CMP and the entropy (fine and coarse) of NP^16^. Studies have shown that NP is considered the most sensitive period for detecting and describing lesions and it is superior to CMP images in lesion detection and superior or equal to CMP images in lesion characterization, which is because the enhancement of the tumor is lower than that of the surrounding renal parenchyma during NP^39^. In our study, CMP showed the equivalent performance to NP when using the SVM classifier, but the NP was overall better than the CMP in terms of model stability. On the other hand, the texture features of the EP were poor in detecting high and low nuclear grade tumors, probably because the differences in enhancement rates partially disappeared as the uptake of the contrast agent continued in the slowly enhancing tumors.

We also find that the classification performance of deep learning model is equivalent to that of machine learning model, even worse than that of machine learning model. On the one hand, due to the lack of data, on the other hand, it is insensitive to the subtle changes in the image between multiple enhancement periods. The main limitation of our study is that the dataset is relatively small due to the strict inclusion criteria, which required the dataset to contain three stages. However, splitting the independent test set from the small sample size dataset will further exacerbate the data scarcity problem, not only that, our research intention is not to build a model that may have higher classification accuracy, but to conduct a comparative evaluation and analysis of the three stages. At the same time, in order to reduce the result bias caused by the small dataset, we conducted extensive internal verification by setting multiple oversampling random seed points, and obtained consistent and stable results, which further increased the reliability of our results. Besides, this study is retrospective, and prospective studies are warranted in the future to further validate the performance of these models in distinguishing high- and low-grade nuclear grades at different stages. Besides, dataset 2 is an external independent verification set. We verified the optimal machine learning model and deep learning model that we trained from dataset 1 on dataset 2. Both the intermediate process data (such as the characteristics of extraction and screening, see the Supplementary Table) and the final results are consistent with our results on dataset 1, with only a small range of errors, which we think are within the allowable range of experiments on different data sets.

At present, the machine learning classification algorithm is mainly applied to CT images, and the medical image data used in this study is limited to CT images. However, if the patient's pathological images and other modal data such as gene sequencing can be included at the same time, more comprehensive disease characteristics and biomarkers can be obtained. A multi-modal fusion model can be designed for multi-stage diagnosis of pathological nuclear grading of ccRCC, which will help to reveal the correlation between different modal data and the potential biological mechanism. So as to better utilize the complementarity and correlation between these data and provide more comprehensive and credible auxiliary information for clinical diagnosis, like the presence of necrosis, central scar presence, renal sinus, and upper urinary tract involvement and extrarenal extension.

## Conclusion

In conclusion, we compared and explored the pros and cons of different phases based on ccRCC multiphase contrast-enhanced CT scan images in distinguishing high nuclear-grade and low nuclear-grade, and the results showed that the nephrography phase was the best period. Firstorder_Kurtosis and firstorder_90Percentile feature play a vital role in the classification task.

This conclusion has important reference value for clinicians to make preoperative evaluation and make treatment plans, and is of great significance to improve the early screening rate of renal cell carcinoma and improve the prognosis of patients.

### Supplementary Information

Supplementary Tables.

## Data Availability

The code can be found in the Supplementary Material. Data available on request due to privacy and ethical restrictions.
