# Supplementary material for: Multiphase comparative study for WHO/ISUP nuclear grading diagnostic model based on enhanced CT images of clear cell renal cell carcinoma
Source: Sci Rep. 2024 May 27;14:12043. doi: 10.1038/s41598-024-60921-x (PMC11130204; doi:10.1038/s41598-024-60921-x)
Supplement: Supplementary file 1 — Supplementary Tables. [file 41598_2024_60921_MOESM1_ESM.docx]

Supplementary Material

**Title：**Multiphase comparative study for WHO/ISUP nuclear grading diagnostic model based on enhanced CT images of clear cell renal cell carcinoma

Author list：Chenyang Lu^1^, Yangyang Xia^2†^, Jiamin Han^1^, Wei Chen^3^, Xu Qiao^1, 4^*, Rui Gao^1^*, and Xuewen Jiang^2^*

# Supplementary Figures and Tables

**Table 1** The whole feature of extraction.

| firstorder_10Percentile | glcm_JointAverage | glrlm_ShortRunHighGrayLevelEmphasis |
| --- | --- | --- |
| firstorder_90Percentile | glcm_JointEnergy | glrlm_ShortRunLowGrayLevelEmphasis |
| firstorder_Energy | glcm_JointEntropy | glszm_GrayLevelNonUniformity |
| firstorder_Entropy | glcm_MaximumProbability | glszm_GrayLevelNonUniformityNormalized |
| firstorder_InterquartileRange | glcm_SumEntropy | glszm_GrayLevelVariance |
| firstorder_Kurtosis | glcm_SumSquares | glszm_HighGrayLevelZoneEmphasi |
| firstorder_Maximum | gldm_DependenceEntropy | glszm_LargeAreaEmphasis |
| firstorder_Mean | gldm_DependenceNonUniformity | glszm_LargeAreaHighGrayLevelEmphasis |
| firstorder_MeanAbsoluteDeviation | gldm_DependenceNonUniformityNormalized | glszm_LargeAreaLowGrayLevelEmphasis |
| firstorder_Median | gldm_DependenceVariance | glszm_LowGrayLevelZoneEmphasis |
| firstorder_Minimum | gldm_GrayLevelNonUniformity | glszm_SizeZoneNonUniformity |
| firstorder_Range | gldm_GrayLevelVariance | glszm_SizeZoneNonUniformityNormalized |
| firstorder_RobustMeanAbsoluteDeviation | gldm_HighGrayLevelEmphasis | glszm_SmallAreaEmphasis |
| firstorder_RootMeanSquared | gldm_LargeDependenceEmphasis | glszm_SmallAreaHighGrayLevelEmphasis |
| firstorder_Skewness | gldm_LargeDependenceHighGrayLevelEmphasis | glszm_SmallAreaLowGrayLevelEmphasis |
| firstorder_TotalEnergy | gldm_LargeDependenceLowGrayLevelEmphasis | glszm_ZoneEntropy |
| firstorder_Uniformity | gldm_LowGrayLevelEmphasis | glszm_ZonePercentage |
| firstorder_Variance | gldm_SmallDependenceEmphasis | glszm_ZoneVariance |
| glcm_Autocorrelation | gldm_SmallDependenceHighGrayLevelEmphasis | shape_Elongation |
| glcm_ClusterProminence | gldm_SmallDependenceLowGrayLevelEmphasis | shape_Flatness |
| glcm_ClusterShade | glrlm_GrayLevelNonUniformity | shape_LeastAxisLength |
| glcm_ClusterTendency | glrlm_GrayLevelNonUniformityNormalized | shape_MajorAxisLength |
| glcm_Contrast | glrlm_GrayLevelVariance | shape_Maximum2DDiameterColumn |
| glcm_Correlation | glrlm_HighGrayLevelRunEmphasis | shape_Maximum2DDiameterRow |
| glcm_DifferenceAverage | glrlm_LongRunEmphasis | shape_Maximum2DDiameterSlice |
| glcm_DifferenceEntropy | glrlm_LongRunHighGrayLevelEmphasis | shape_Maximum3DDiameter |
| glcm_DifferenceVariance | glrlm_LongRunLowGrayLevelEmphasis | shape_MeshVolume |
| glcm_Id | glrlm_LowGrayLevelRunEmphasis | shape_MinorAxisLength |
| glcm_Idm | glrlm_RunEntropy | shape_Sphericity |
| glcm_Idmn | glrlm_RunLengthNonUniformity | shape_SurfaceArea |
| glcm_Idn | glrlm_RunLengthNonUniformityNormalized | shape_SurfaceVolumeRatio |
| glcm_Imc1 | glrlm_RunPercentage | shape_VoxelVolume |
| glcm_Imc2 | glrlm_RunVariance |  |
| glcm_InverseVariance | glrlm_ShortRunEmphasis |  |

**Table 2** The selected features (frequency greater than 15) in NP staging using three classifiers in Dataset1.

| **SVM** | **RF** | **XGB** | | | |  |  |
| --- | --- | --- | --- | --- | --- | --- | --- |
| glcm_Contrast | **firstorder_Skewness** | | glrlm_RunLengthNonUniformityNormalized | | | |  |
| **firstorder_Skewness** | glrlm_RunLengthNonUniformityNormalized | | **firstorder_Skewness** | | | |  |
| shape_Elongation | **glrlm_ShortRunEmphasis** | | **glrlm_ShortRunEmphasis** | | | |  |
| shape_Sphericity | gldm_SmallDependenceHighGrayLevelEmphasis | |  | | | |  |
| glcm_ClusterShade |  | |  | | | |  |
| **glrlm_ShortRunEmphasis** |  | |  | | | |  |
| glrlm_GrayLevelNonUniformityNormalized |  | |  | | | |  |
| glrlm_ShortRunLowGrayLevelEmphasis |  | |  | | | |  |
| firstorder_Minimum |  | |  | | | |  |
| glcm_Imc1 |  | |  | | | |  |
| glszm_SmallAreaLowGrayLevelEmphasis |  | |  | | | |  |
| glszm_SmallAreaHighGrayLevelEmphasis |  | |  | | | |  |
| glszm_GrayLevelVariance |  | |  | | | |  |
| firstorder_Mean |  | |  | | | |  |
| shape_SurfaceVolumeRatio |  | |  | | | |  |
| glcm_DifferenceVariance |  | |  | | | |  |
| glszm_LargeAreaLowGrayLevelEmphasis |  | |  | | | |  |
| firstorder_InterquartileRange |  | |  | | | |  |
| glszm_LowGrayLevelZoneEmphasis |  | |  |  |  | | |
| Bold in black: the common features selected by the three classifiers. | | | | | |  |  |

**Table 3** The selected features (frequency greater than 15) in CMP staging using three classifiers in Dataset1.

| **SVM** | | **RF** | | **XGB** | |
| --- | --- | --- | --- | --- | --- |
| shape_Maximum2DDiameterColumn |  | glrlm_RunEntropy |  | **glszm_GrayLevelNonUniformityNormalized** |  |
| shape_Maximum2DDiameterRow |  | gldm_SmallDependenceEmphasis |  | shape_Sphericity |  |
| glszm_LargeAreaLowGrayLevelEmphasis |  | glszm_ZonePercentage |  | glszm_SizeZoneNonUniformityNormalized |  |
| **glszm_GrayLevelNonUniformityNormalized** |  | glszm_LargeAreaLowGrayLevelEmphasis |  |  |  |
|  |  | glcm_DifferenceEntropy |  |  |  |
|  |  | glcm_Correlation |  |  |  |
|  |  | **glszm_GrayLevelNonUniformityNormalized** |  |  |  |
|  |  | shape_Sphericity |  |  |  |
|  |  | glszm_SizeZoneNonUniformityNormalized |  |  |  |
|  |  | firstorder_Median |  |  |  |
|  |  | shape_Elongation |  |  |  |
|  |  | firstorder_Minimum |  |  |  |
|  |  | glszm_SmallAreaEmphasis |  |  |  |
|  |  | firstorder_Maximum |  |  |  |
|  |  | firstorder_Range |  |  |  |
| Bold in black: the common features selected by the three classifiers. | | | | | |

**Table 4** The selected features (frequency greater than 15) in EP staging using three classifiers in Dataset1.

| **SVM** | | **RF** | | **XGB** | |
| --- | --- | --- | --- | --- | --- |
| glcm_ClusterProminence |  | glrlm_ShortRunEmphasis |  | gldm_DependenceVariance |  |
| **glcm_Idmn** |  | gldm_DependenceVariance |  | glrlm_ShortRunEmphasis |  |
| shape_Maximum2DDiameterRow |  | glszm_GrayLevelVariance |  | firstorder_90Percentile |  |
| gldm_SmallDependenceLowGrayLevelEmphasis |  | glszm_SmallAreaHighGrayLevelEmphasis |  | gldm_SmallDependenceLowGrayLevelEmphasis |  |
| glszm_LowGrayLevelZoneEmphasis |  | **glcm_Idmn** |  | **glcm_Idmn** |  |
| glszm_GrayLevelNonUniformity |  | glszm_GrayLevelNonUniformityNormalized |  | glszm_GrayLevelNonUniformityNormalized |  |
| firstorder_Range |  | glcm_Idn |  |  |  |
| glszm_ZoneEntropy |  | firstorder_RootMeanSquared |  |  |  |
| Bold in black: the common features selected by the three classifiers. | | | | | |

**Table 5** The selected features (frequency greater than 15) in NP staging using three classifiers in Dataset2.

| **SVM** | **RF** | **XGB** |  |
| --- | --- | --- | --- |
| glszm_LargeAreaHighGrayLevelEmphasis | firstorder_10Percentile | firstorder_10Percentile | |
| **shape_Elongation** | firstorder_Kurtosis | firstorder_Skewness | |
| glcm_MaximumProbability | firstorder_Skewness | firstorder_90Percentile | |
| glszm_GrayLevelNonUniformityNormalized | **shape_Elongation** | glszm_GrayLevelNonUniformityNormalized | |
| shape_SurfaceVolumeRatio | firstorder_Mean | shape_Sphericity | |
|  |  | firstorder_Kurtosis | |
|  |  | **shape_Elongation** | |
|  |  | glcm_ClusterShade | |
|  |  | firstorder_Maximum | |
|  |  | firstorder_Mean | |
|  |  | firstorder_Energy | |
|  |  | glcm_Id | |
|  |  | firstorder_InterquartileRange | |
|  |  | firstorder_Entropy | |
|  |  | glszm_LargeAreaHighGrayLevelEmphasis | |
|  |  | firstorder_MeanAbsoluteDeviation | |
|  |  | shape_MinorAxisLength | |
|  |  | firstorder_Minimum | |
|  |  | glszm_SmallAreaLowGrayLevelEmphasis | |
|  |  | shape_Maximum2DDiameterColumn | |
|  |  | glszm_LowGrayLevelZoneEmphasis | |
|  |  | glszm_LargeAreaEmphasis | |
| Bold in black: the common features selected by the three classifiers. | | |  |

**Table 6** The selected features (frequency greater than 15) in CMP staging using three classifiers in Dataset2.

| **SVM** | **RF** | **XGB** |  |
| --- | --- | --- | --- |
| glszm_SmallAreaLowGrayLevelEmphasis | firstorder_10Percentile | **firstorder_Median** | |
| glszm_GrayLevelNonUniformityNormalized | firstorder_90Percentile | glcm_ClusterShade | |
| firstorder_Kurtosis | firstorder_Energy | shape_Flatness | |
| gldm_SmallDependenceHighGrayLevelEmphasis | firstorder_Entropy | firstorder_Skewness | |
| glszm_LowGrayLevelZoneEmphasis | firstorder_InterquartileRange | glszm_SmallAreaEmphasis | |
| **firstorder_Median** | firstorder_Kurtosis | glszm_SmallAreaLowGrayLevelEmphasis | |
| glrlm_LowGrayLevelRunEmphasis | firstorder_Maximum | glcm_Imc1 | |
| shape_Sphericity | firstorder_Mean | shape_Elongation | |
|  | firstorder_MeanAbsoluteDeviation | glszm_GrayLevelVariance | |
|  | **firstorder_Median** | glcm_InverseVariance | |
|  | firstorder_Minimum | shape_LeastAxisLength | |
|  | firstorder_Range | glrlm_LongRunHighGrayLevelEmphasis | |
|  | firstorder_RobustMeanAbsoluteDeviation |  | |
|  | firstorder_RootMeanSquared |  | |
|  | firstorder_Skewness |  | |
|  | firstorder_TotalEnergy |  | |
| Bold in black: the common features selected by the three classifiers. | | |  |

**Table 7** The selected features (frequency greater than 15) in EP staging using three classifiers in Dataset2.

| **SVM** | **RF** | **XGB** |  |
| --- | --- | --- | --- |
| firstorder_10Percentile | shape_Elongation | glcm_Autocorrelation | |
| glrlm_RunEntropy | shape_Flatness | shape_Flatness | |
| glszm_GrayLevelVariance | glcm_Autocorrelation | glcm_Idmn | |
| glszm_GrayLevelNonUniformityNormalized | gldm_HighGrayLevelEmphasis | glcm_MaximumProbability | |
| glszm_GrayLevelNonUniformity | shape_MajorAxisLength | shape_MajorAxisLength | |
| glrlm_ShortRunLowGrayLevelEmphasis | firstorder_90Percentile | firstorder_Kurtosis | |
| **glrlm_ShortRunHighGrayLevelEmphasis** | gldm_DependenceNonUniformityNormalized | **glrlm_ShortRunHighGrayLevelEmphasis** | |
| glrlm_RunVariance | firstorder_10Percentile | glrlm_RunLengthNonUniformity | |
| glrlm_RunPercentage | glszm_SmallAreaEmphasis | firstorder_90Percentile | |
| glrlm_LowGrayLevelRunEmphasis | glcm_JointAverage | firstorder_Entropy | |
| glszm_LargeAreaEmphasis | **glrlm_ShortRunHighGrayLevelEmphasis** | firstorder_Skewness | |
| glrlm_LongRunLowGrayLevelEmphasis | gldm_LargeDependenceHighGrayLevelEmphasis |  | |
| glrlm_LongRunHighGrayLevelEmphasis | glcm_SumSquares |  | |
| glrlm_LongRunEmphasis | glszm_ZoneVariance |  | |
|  | glszm_SizeZoneNonUniformityNormalized |  | |
|  | glcm_MaximumProbability |  | |
| Bold in black: the common features selected by the three classifiers. | | |  |
